# Anchoring Intrinsically Disordered Proteins to Multiple Targets: Lessons from *N*-Terminus of the p53 Protein

**DOI:** 10.3390/ijms12021410

**Published:** 2011-02-23

**Authors:** Yongqi Huang, Zhirong Liu

**Affiliations:** 1 State Key Laboratory for Structural Chemistry of Unstable and Stable Species, College of Chemistry and Molecular Engineering, Peking University, Beijing 100871, China; 2 Center for Theoretical Biology, Peking University, Beijing 100871, China; 3 Beijing National Laboratory for Molecular Sciences, Peking University, Beijing 100871, China

**Keywords:** anchor residue, intrinsically disordered proteins, binding promiscuity, molecular recognition features, p53, MDM2, Taz2

## Abstract

Anchor residues, which are deeply buried upon binding, play an important role in protein–protein interactions by providing recognition specificity and facilitating the binding kinetics. Up to now, studies on anchor residues have been focused mainly on ordered proteins. In this study, we investigated anchor residues in intrinsically disordered proteins (IDPs) which are flexible in the free state. We identified the anchor residues of the *N*-terminus of the p53 protein (Glu17–Asn29, abbreviated as p53N) which are involved in binding with two different targets (MDM2 and Taz2), and analyzed their side chain conformations in the unbound states. The anchor residues in the unbound p53N were found to frequently sample conformations similar to those observed in the bound complexes (*i.e.*, Phe19, Trp23, and Leu26 in the p53N-MDM2 complex, and Leu22 in the p53N-Taz2 complex). We argue that the bound-like conformations of the anchor residues in the unbound state are important for controlling the specific interactions between IDPs and their targets. Further, we propose a mechanism to account for the binding promiscuity of IDPs in terms of anchor residues and molecular recognition features (MoRFs).

## 1. Introduction

Proteins are the machines of living systems, and their interaction with other molecules is a central step to perform functions. Considerable efforts have been devoted to understanding the principles governing protein–protein interactions, including interface contacts, morphology, residue conservation, and secondary structures [1–5]. In general, the complex interface is not flat, and some residues from one protein deeply insert into the binding groove of the partner, resulting in the greatest changes in the solvent accessible surface area (SASA) among all the interface residues. Such residues are called anchor residues [6]. Anchor residues have been extensively studied, and their critical roles in specific molecular recognition processes have been widely addressed [6–12]. The most remarkable feature of anchor residues is their conformational preference in the unbound state. All-atom molecular dynamics (MD) simulations have shown that anchor residues in the unbound state are in conformations similar to those observed in the bound complexes [6,7]. The conformational preference of the anchor residues has been successfully applied to improve docking efficiency [9,10]. Recently, Csermely *et al.* [13] figured out an extended view of binding which embraces a repertoire of conformational selection and structural adjustment process, where they highlighted the important role of anchor residues in the binding process. In their mechanism, conformational selection of the anchor residues in the transient encounter process is critical in the stabilization of the encounter complexes due to their large surface area. The encounter complexes then undergo further induced-fit to complete the binding event [14].

Up to now, studies on anchor residues have been restricted to ordered proteins, *i.e.*, proteins that can be described by defined three-dimensional structures. However, not all proteins form unique structures in the free state. There exists another special family of proteins—the intrinsically disordered proteins (IDPs)—which are flexible in the free state and should be described by an ensemble of conformations [15–30]. The sequence composition of IDPs is very different from that of ordered proteins, and therefore IDPs can be reliably predicted through bioinformatics studies. More than 60 computational tools have been developed for disorder prediction and many of them have been reviewed in reference [31]. IDPs are enriched in cellular functions, such as signaling transduction and transcription regulation [26,32,33], and conformational flexibility is extremely important for IDPs to interact with their targets [34–38]. In experiments, NMR techniques, small-angle X-ray scattering, and different spectroscopic and hydrodynamic methods have been widely used to elucidate the structural features of IDPs [39–43], including the molecular sizes, secondary structural elements, coupled folding-binding processes, and aggregation propensities. In particular, combined with experimentally determined restraints, computer simulations have provided the ensemble-level pictures of IDPs [40].

Experimental and simulation studies have provided evidence that specific functional regions of IDPs are spatially exposed and may be the primary contact sites in the binding processes [44]. Through analysis of complex structures and disorder predictions, short binding regions within long disordered sequences were identified and termed as molecular recognition features (MoRFs) [45–48]. MoRFs differ from other disordered regions due to their significant secondary structure propensities, and may possess preformed structures similar to those in the complex state [49–52]. To form the complexes, IDPs use much of their surface to form the interface [47,53,54]; some residues of IDPs insert deeply into the binding partners [47,55]. Consequently, these highly buried residues can also be defined as anchor residues as those defined in ordered proteins. Although the overall structures of MoRFs have been extensively characterized [52,56,57], an atomic-level examination of the MoRFs in terms of anchor residues is still missing. In addition, IDPs are implicated in multiple interactions as their structural plasticity allows them to efficiently interact with different targets. So a residue may be an anchor residue when binding to one target, whereas in a different protein complex this same residue may not be an anchor. Therefore, it would be of significant importance to investigate the behaviors of anchor residues when IDPs bind to different targets.

In this study, we performed all-atom MD simulations on a helical region of the *N*-terminal transactivation domain (TAD) of the p53 protein (Glu17–Asn29) (abbreviated as p53N hereafter). p53 is a transcription factor and is critical in preventing cancer development. The p53N region is the binding site of multiple targets, such as MDM2, MDMX, CBP/p300, Taz2, and Bcl-X_L_ [58]. Structure analysis has shown that the p53N region is highly disordered with a transient helix structure formed within residues Thr18–Leu26 [59–61]. When binding to its targets, e.g., MDM2, the p53N helix is prolonged and stabilized [62,63]. Therefore, p53N is a MoRF with a preformed helical structure that binds to MDM2. Using p53N as an example of IDPs, we analyzed the side chain conformations of anchor residues in the unbound state, and compared their conformations with those in the p53N-MDM2 and p53N-Taz2 complexes to address the role of anchor residues in the molecular recognition processes.

## 2. Results and Discussion

### 2.1. Anchor Residues in the p53N Complexes

**Figure 1.**
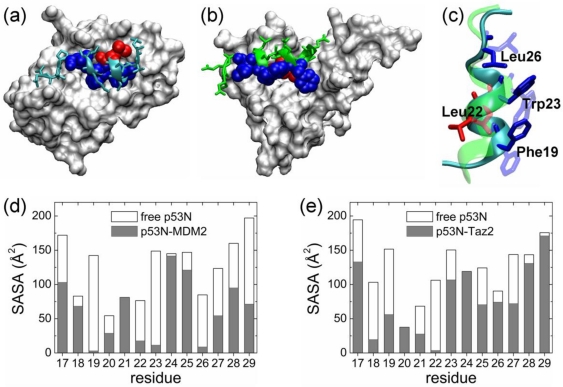
The analysis of the structures of p53N when bound to different targets. (**a**,**b**) Complex structures of the p53N-MDM2 complex (**a**) and p53N-Taz2 complex (**b**). (**c**) Structure superposition of p53N from p53N-MDM2 and p53N-Taz2 based on the backbone RMSD. In (**a**–**c**), the MDM2 and Taz2 are presented as surface, whereas the p53N is presented as ribbon. Phe19, Trp23, and Leu26 are shown as blue balls in (**a**,**b**) and sticks in (**c**), Leu22 is shown in red, whereas other residues of the p53N are colored cyan (when in complex with MDM2) or green (when in complex with Taz2). (**d**,**e**) SASA analysis of the p53N in complex with MDM2 (**d**) and Taz2 (**e**).

p53N is versatile and interacts with multiple targets. The binding profile of p53N in the p53N-MDM2 complex was different from that in the p53N-Taz2 complex (Figure 1). In the p53N-MDM2 complex, Phe19, Trp23, and Leu26 were highly buried in the binding groove (Figure 1a,d). In contrast, in the p53N-Taz2 complex, only Leu22 was highly buried (Figure 1b,e). Structural superposition showed that p53N used different surfaces of a helix structure to bind to these two targets (Figure 1c), *i.e.*, the p53N helix rotated about 90° in these complexes. So the buried residues in the p53N-MDM2 complex (Phe19, Trp23, and Leu26) were exposed to solvent in the p53N-Taz2 complex (Figure 1a,b). In globular protein complexes, anchor residues are identified based on the structure of the complexes. They correspond to solvent exposed residues that are fully buried upon binding to a target, yielding the largest change in SASA [6]. However, for complexes formed by IDPs, the determination of SASA for the unbound state may not be so straightforward, since an ensemble of conformations instead of a unique conformation are needed to describe the unbound state of an IDP. We calculated the SASA for the unbound p53N by two different approaches. The simplest one was removing the Taz2 and MDM2 proteins from the p53N-Taz2 and p53N-MDM2 complexes and then calculating the SASA of the remaining p53N (Figure 1d,e). We further calculated the SASA using the simulated conformations of p53N (Figure S1). In general, different approaches gave similar results. According to the initial definition of anchor residues [6], we suggested that anchor residues of IDPs should expose to solvent in the unbound state and become fully buried (SASA ≤ 15 Å^2^ was used here) after binding. Therefore, we identified Phe19, Trp23, and Leu26 as the anchor residues in the complex with MDM2, and Leu22 as the anchor residue in the complex with Taz2. It was noted that the side chain conformations of Phe19, Leu22, Trp23, and Leu26 in the MDM2 complex were remarkably different from those in the Taz2 complex (Figure 1c). Consistent with conformation ensemble of the p53 TAD [64], Phe19, Leu22, Trp23, and Leu26 were not buried in our simulated unbound states.

### 2.2. Transient Stable Helix of the p53N

**Figure 2.**
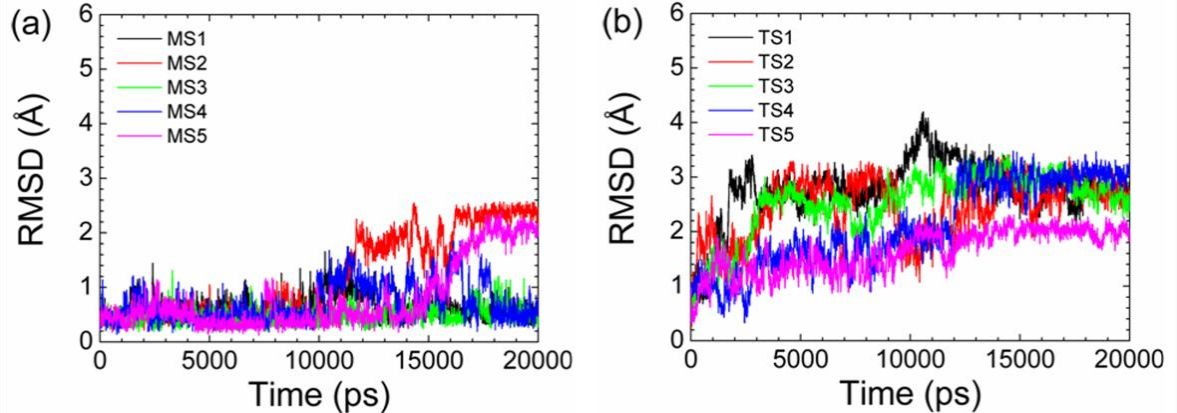
RMSD relative to the backbone of the helical region (Phe19–Leu26) for p53N from p53N-MDM2 (**a**) and from p53-Taz2 (**b**) during the simulations. Simulation trajectories MS1–MS5 and TS1–TS5 are presented.

Although the p53N region is rather flexible in solution, experiments indicate that residues Thr18–Leu26 form a transiently stable helix which will be further stabilized in the complex state [59]. To study the conformational preference of unbound p53N, we conducted multiple simulations on the p53N with initial conformations adopted from the p53N-MDM2 and p53N-Taz2 complexes under identical conditions (see the *Method Section* for details). The helical structures were only transiently stable and unfolded in the simulations under 300 K (Figures 2 and S2). We defined the unfolding time of each trajectory through secondary structure analysis, RMSD relative to the initial helical structure, and inspection of the structures (with detailed results in Table S1). The average unfolding time was 24.5 ± 11.8 ns for the helix from p53N-MDM2 complex and 5.2 ± 3.1 ns for the helix from p53N-Taz2 complex. Clearly, the helix from the p53N-MDM2 complex was much more stable than that from the p53N-Taz2 complex. This may be due to the deformation of the second turn (in the *C*-terminus) of the p53N helix from the p53N-Taz2 complex, because the unfolding of the p53N helix from either p53N-MDM2 or p53N-Taz2 usually started from the deformation of the second turn during the simulations. We also compared the properties of our ensemble with those from the Daughdrill’s group [64]. Consistent with their results, the distributions of amide nitrogen distances between residues *i:i* + 5 were bimodal, and Phe19–Trp23 showed greater probabilities of collapsed structures (data not shown).

### 2.3. Analysis of the Side Chain Conformations of the Anchor Residues

**Figure 3.**
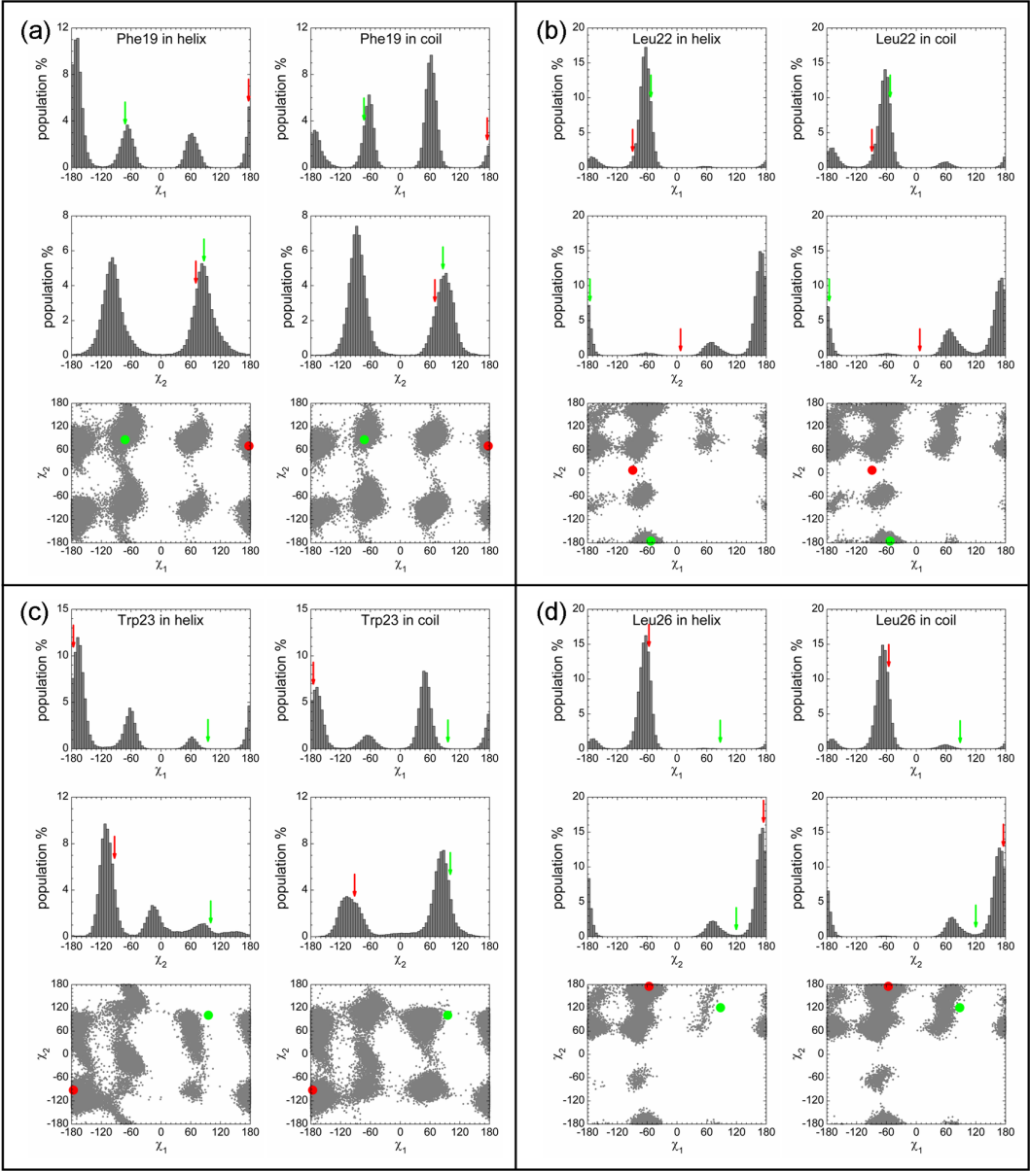
Conformational analysis of the anchor residues in the helix state and disordered state of the unbound p53N. The bound conformation values in the p53N-MDM2 and p53N-Taz2 complexes are denoted by red and green markers, respectively.

Since the helix of p53N is transiently stable, it is expected that the free energy of the helix state in the free form is higher than the disordered state (Figure S3). The free energy barrier of unfolding is lower than the free energy barrier of folding, resulting in a greater unfolding rate than a folding rate. To obtain conformations of the helix state, we separated the p53N helix from the complex state and carried out simulations. The system quickly relaxed to the free energy basin of the helix state and probably got equilibrium in the basin before it unfolded to the disordered state (Figure 2). To obtain conformations of the disordered state, randomly selected disordered structures were used as initial states from simulations. Then the system got equilibrium in the free energy basin of the disordered state. No disorder-to-helix transition was observed in simulations. It was noted that this strategy did not produce an equilibrium population between the helix state and the disordered state, although it gave the distribution of the side chain conformations within each state (Figure 3).

We analyzed the side chain conformations for the helix state and the disordered state. In our simulations, the helix of p53N from the p53N-Taz2 complex unfolded very quickly (∼5 ns). Within such a short period, conformational sampling of the side chains in the helix state was insufficient. So we analyzed the side chain conformations based on the simulations of p53N from the p53N-MDM2 complex which has a much longer unfolding time. The conformational sampling was found to be more efficient in this case. For example, more than 130 transitions were observed between the two main χ_2_ conformations of Phe19 and the distribution of the χ^2^ of Phe19 appeared to be perfectly symmetric (Figure 3a) which is required due to the symmetric nature of Phe.

In this study, we considered two binding targets, so for the four identified anchor residues, each has two bound-like conformations: one is an anchor-type (conformations of Phe19, Trp23, and Leu26 in the p53-MDM2 complex, and that of Leu22 in the p53-Taz2 complex); the other is a non-anchor conformation (conformations of Phe19, Trp23, and Leu26 in the p53-Taz2 complex, and that of Leu22 in the p53-MDM2 complex). Remarkably, the analysis showed that the anchor residues dominantly sampled the anchor-type bound-like conformations rather than the non-anchor bound-like conformations regardless of whether the helix or the disordered states were examined (Figure 3 and Table 1). The anchor residue with the highest population of the non-anchor bound-like conformation was Phe19, but its value was only 12.2% and 6.0% in the helical and disordered states, much smaller than the corresponding value of the anchor-type conformation (59.5% and 19.2%). For the other three anchor residues, the population of non-anchor bound-like conformation was negligible. It was also noted that the formation of a (transient) helical structure enhanced the predominance of the anchor-type bound-like conformations. In the p53N-MDM2 complex, χ_1_ and χ_2_ of Phe19 are 177° and 71°. The population of this (anchor-type) rotamer increased by a factor of ∼2 (*i.e.*, 19.2% *vs.* 59.5%, Table 1), when the p53N transformed from a disordered state to a helix state. Trp23 showed a similar trend. Although the extent of the increase was weaker for Leu22 and Leu26, the formation of a helix still increased the population of the anchor-type conformations.

Although the initial structure for simulations was isolated from the p53N-MDM2 complex, Leu22 sampled conformations similar to that in the p53N-Taz2 complex but not similar to that in the p53N-MDM2 complex (Figure 3b and Table 1). Consequently, the discrimination between the populations of the anchor-type and non-anchor bound-like conformations was not caused by a bias of the initial states. It could not be solely explained in terms of the side-chain rotamer preferences either: rotamer library data [65] indicated that the rotamer of Phe19 preferred the non-anchor bound-like conformation (47.08%) rather than the anchor-type (31.71%); however, the trend was reversed in the helix state (12.2% *vs.* 59.5%). The preference of Trp23 on the anchor-type conformation was low in the rotamer library (16.21%), which was greatly enhanced in the helix state (62.4%). These observations suggest that the preference of the anchor-type conformations is intrinsic to the transient helical structure of the unbound p53N.

**Figure 4.**
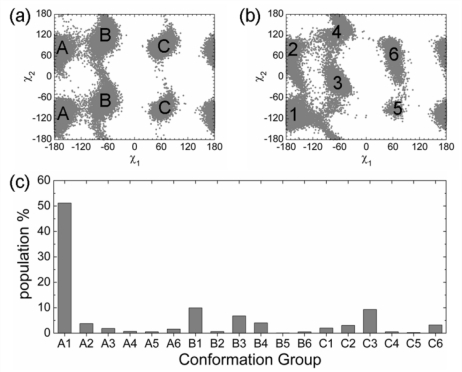
Combined analysis of the conformations of Phe19 and Trp23 in the helix state. (**a**) Side chain dihedral distribution of Phe19; (**b**) Side chain dihedral distribution of Trp23; (**c**) The combination of conformations of Phe19 and Trp23.

Figure 3 and Table 1 show that the extent of the population shift during the helix-disorder transition was similar for Phe19 and Trp23, suggesting synchronous dynamics between these residues. χ_1_ of Phe19 sampled three regions (labeled as A, B, and C in Figure 4a. Due to the symmetric nature of Phe, χ_2_ was not distinguished.). In the χ_1_–χ_2_ space, Trp23 sampled six regions (labeled from 1 to 6 in Figure 4b). So there were 18 possible combinations of the conformations for Phe19 and Trp23, where the A1 group was the anchor-type bound-like conformation corresponding to that in the p53N-MDM2 complex. The population analysis showed a remarkable feature that the population of the A1 group (51%) was significantly higher than all other possible combinations (Figure 4c). Furthermore, the population of the A1 group was very close to the individual population of the anchor-type bound-like conformations of Phe19 and Trp23 (59.5% and 62.4%, respectively), showing a strong correlation between these two residues. In the helical conformation of p53N, Phe19 and Trp23 drove (or confined) each other to the anchor-type bound-like conformations.

**Figure 5.**
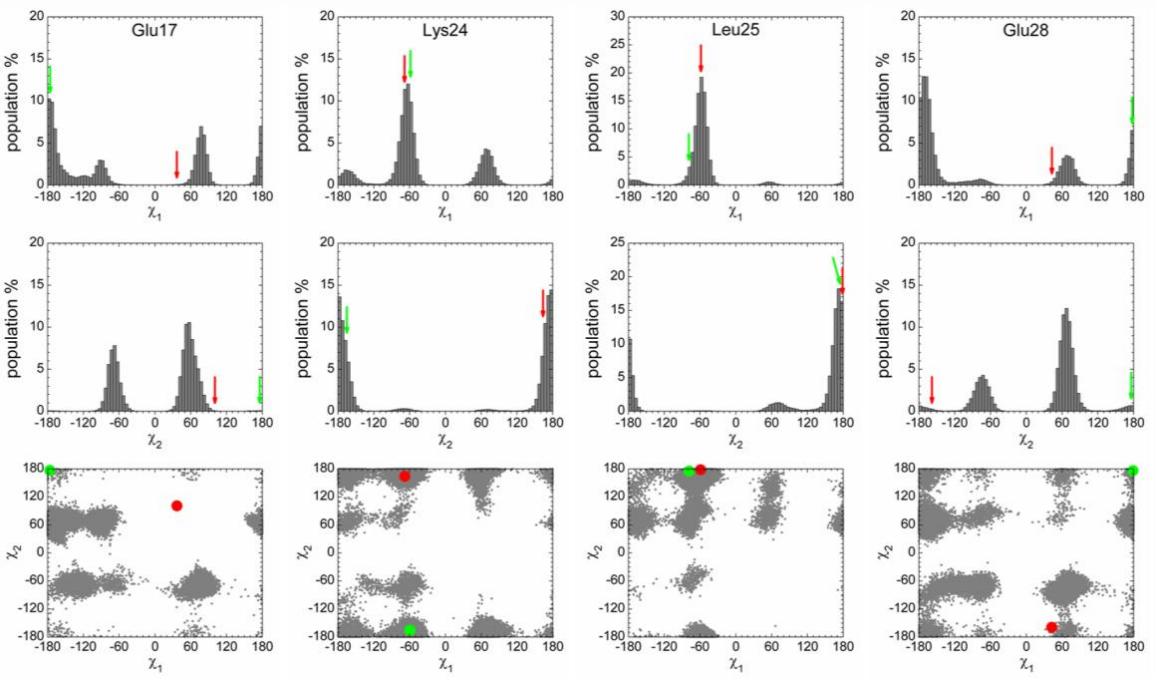
Conformational analysis of non-anchor residues in the helix state. Bound conformations in the p53N-MDM2 and p53N-Taz2 complexes are denoted by red and green markers, respectively.

We also analyzed the side chain conformations of non-anchor residues, e.g., Glu17, Lys24, Leu25, and Glu28 (Figure 5). For Glu17 and Glu28, both bound conformations (in the p53N-MDM2 and p53N-Taz2 complexes) were rarely sampled in the helix and disordered states (data not shown) during the simulations. Therefore, the conformations of these residues in the complex states were induced by interactions with the targets. For Lys24 and Leu25, bound-like conformations were frequently sampled in the simulations and the formation of the helix increased the populations.

### 2.4. Transient Formation of Helical Structures Promotes the Binding Process

**Figure 6.**
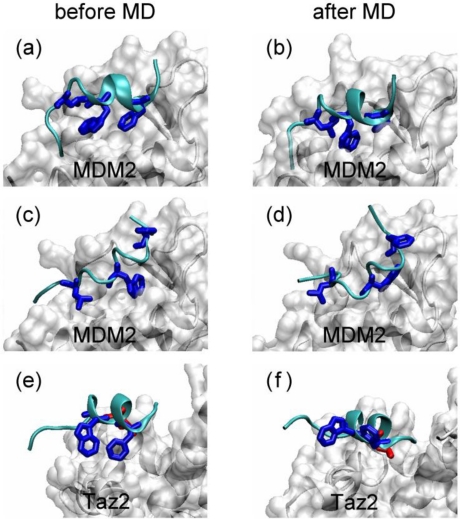
The roles of anchor residue conformations in the evolution of the transient encounter complex towards the bound state. (**a**,**b**) p53N in a bound-like conformation; (**c**,**d**) p53N in a distorted conformation; (**e**,**f**) p53N in a bound-like conformation. p53N is shown as cyan ribbons and the binding targets are shown as surface. Phe19, Trp23, and Leu26, are shown as blue sticks. Leu22 is shown as red sticks.

Because atomic information of the MoRFs is missing and how/why MoRFs and anchor residues initiate the binding process is unclear. To identify the role of anchor residues and the performed structure of MoRFs in the binding processes, we performed binding simulations of p53N to MDM2 and Taz2. Firstly, we randomly selected five conformations from the A1 group (Figure 4) as initial conformations of the p53N. Then, we placed the p53N close to the binding groove of MDM2 with the correct orientation (Figure 6a). This was to mimic the encounter of the two proteins. In these encounter states, the anchor residues did not insert into the binding groove. Based on these *in silico* encounter states, we conducted MD simulations to track the evolution process. As expected, after the local conformational rearrangements on the binding groove of MDM2, Phe19, Trp23, and Leu26 inserted into the binding groove within ∼1 ns (Figure 6b). This result is consistent with the observations of binding the native p53N to MDM2 [66], because conformations in the A1 group were native-like. Our results confirm the validity of results which are based on the prerequisite that p53N is in a bound conformation during the encounter process [66,67]. Furthermore, we also performed simulations with the p53N in other conformations, e.g., conformations from the B3 group and the disordered states. Within 10 ns of simulations, p53N did not evolve towards the bound conformations but formed non-native interactions with MDM2 (Figure 6c,d). The same conformations of p53N above were also used to simulate binding of p53N to Taz2. For p53N in the helix state (*i.e.*, conformations from the A1 group and B3 group), Leu22 inserted into the binding groove quickly; however, the correct conformations of Phe19 and Trp23 were not observed within 10 ns (Figure 6e,f). This was due to the steric constraints at the binding interface. Therefore, induced formation of the correct conformations of Phe19 and Trp23 may take longer. For p53N in the disordered state, only non-native interactions between p53N and Taz2 were observed.

Although experimental and computational studies have shown that MDM2 undergoes structural rearrangement, in particular in the binding groove, upon the p53N binding [66,68], it does not contradict the concept of a preformed bound-like conformation of the unbound p53N. On the contrary, the bound-like conformation of p53N in the unbound state actually promotes the groove opening of the unbound MDM2 [66]. Furthermore, the important roles of Phe19, Trp23, and Leu26 in the binding process of p53N with MDM2 and Leu22 in the binding process of p53N with Taz2 have been studied thermodynamically [69,70]; however, their roles in the kinetic process is not clear. In this work, through simulations, we found that preformed bound-like conformations of these anchor residues promoted the binding process.

### 2.5. Discussions: Roles of Anchor Residues in Molecular Recognition

In this study, we tried to extend the concept of anchor residues to IDPs and understand the conformational properties of the anchor residues within a highly flexible context. To this end, we performed atomic MD simulations on an extensively studied system, the p53N region. In the unbound state, p53N is rather flexible; however, once the helix is partially and transiently formed, simulations showed that the anchor residues were restricted to their anchor-type bound-like conformations and were primed for interacting with their targets (Figures 3 and 4, and Table 1).

A comparison of the bound conformations of the anchor residues and non-anchor residues with the rotamer library derived from the Protein Data Bank [65] further supports the concept that anchor residues adopt preformed bound-like conformations (Table 1). In the p53N-MDM2 complex, the conformations of the anchor residues (Phe19, Trp23, and Leu26) represent the major conformations in the rotamer library, whereas the conformations of Glu17, Leu22, and Glu28 exhibit very low values in the rotamer library. Conversely, in the complex p53N-Taz2, the conformation of Leu22 is the major conformation in the library, whereas the conformations of Trp23 and Leu26 exhibit very low populations. The formation of rare rotamers is induced by interactions between p53N and the targets [71].

Significant efforts have been made to identify the structure of MoRFs and understand the molecular recognition processes between IDPs and their binding targets. Experimental and computational studies have shown that preformed structures of MoRFs in the unbound state resemble structures in the bound complexes and therefore facilitate the recognition processes [49,51,52,57,59,72–75]. However, the roles of MoRFs in the molecular recognition processes remain elusive. In this study, we extended the understanding through an investigation of the correlation between the side chain conformations and the overall structure of MoRFs. We suggest that, as in globular proteins, anchor residues also exist in IDPs and are important in the specific molecular recognition processes. Since the side chain conformations are backbone dependent, the preformed structure of MoRFs in the unbound state provides a constraint on the side chain conformations to produce bound-like anchor residues. A recent study showed that the conformational preference of residues in a disordered 20-mer peptide was closely correlated to inhibitory activity [76]. Recently, Kjaergaard *et al.* determined the core structure of a molten globule by NMR and found that the side chain of some hydrophobic residues had preferred rotamers and made specific interactions [77]. Characterization of the MoRFs of Sendai virus nucleoprotein also showed that the transient formation of a helix optimized the interaction with the negatively charged cleft on the surface of the phosphoprotein PX domain [73]. These results support the concept that particular key residues in IDPs have preferred (function-related) conformations in the unbound state.

Combined with the concepts of MoRFs and anchor residues, a feasible mechanism of the recognition process between a disordered binding region and its target emerges. For example, in the binding process of p53 to MDM2, the binding is initiated by an encounter between the preformed bound-like MoRF of p53 and MDM2, which will produce a transient encounter complex. Insertion of the preformed bound-like anchor residues (Phe19, Trp23, and Leu26), which are located on the MoRF, into the binding groove of MDM2 stabilizes the transient encounter complex (Figure 6a,b). Further folding of the backbone and induced-fit of the side chains take place to finally form the native complex. Therefore, in this mechanism, a binding process between an IDP and its target is initiated by a conformational selection and then proceeds by folding upon binding [51,75]. In the simulated binding process of proline-rich motifs to SH3 domains, electrostatic interactions guide the diffusion to form a nonspecific encounter complex state which is stabilized by subsequent anchoring of an arginine of the peptide into the negatively charged groove of the SH3 domain [78]. This gives some support to our proposed mechanism.

To account for the specific recognition process, various mechanisms have been proposed, including the lock-and-key model [79], the induced-fit mechanism [80], and the conformational selection model [81,82]. Recently, Boehr *et al.* proposed a general mechanism constituted by a primary conformational selection event followed by an induced-fit of side chains and the backbone to account for the role of dynamics in the biomolecular recognition process [83]. Similarly, Csermely *et al.* figured out an extended view of binding which embraces a repertoire of selection and adjustment processes [13]. All these mechanisms mainly focus on ordered proteins and the discussions on IDPs are very limited. Our study adds important insights into the molecular recognition mechanisms and extends them to molecular interactions involving IDPs.

IDPs have been proposed to have the ability to bind to multiple targets [38] and p53 is a typical example. The *N*-terminus of p53 binds MDM2, MDMX, Taz2, and Bcl-X_L_, while the *C*-terminus of p53 interacts with S100ββ, Sirtuin, CBP, and Cyclin A2 [58]. SASA analysis shows that p53N uses different anchor residues to bind to different targets (Figure 1). Forming a complex with MDM2, Phe19, Trp23, and Leu26 are the anchor residues, whereas binding with Taz2, Leu22 is the anchor residue. By analyzing the complex structures, Oldfield *et al.* found that the same residues from the *C*-terminus of p53 are used to a different extent in binding to different targets and interactions involving the same residue may exclude each other in different complexes [55]. Our simulations showed that, in the helix state of unbound p53N, Phe19, Trp23, and Leu26 significantly sampled conformations similar to those in the complex with MDM2, whereas Leu22 sampled conformations similar to that in the complex with Taz2 (Figures 3 and 4, Table 1); these indicate a new mechanism to account for the binding promiscuity of IDPs. It is possible that all the anchor residues in the MoRFs frequently sample the bound conformations of the corresponding targets and that a particular target selects a particular group of anchor residues in the binding process. This binding mechanism is advantageous in smoothing molecular interactions [84], and reconciles the binding promiscuity and binding kinetics in the binding process and provides a clearer picture for the one-to-many signaling processes where flexibility has been considered as the main source [55,85,86]. A similar mechanism has been proposed to interpret the structural basis of pregnane X receptor binding promiscuity, where the pregnane X receptor has five hot spot regions and, depending on their sizes and shapes, individual PXR ligands extend into two, three, or four hot spot regions [87].

To extend the mechanism obtained from p53N to other IDPs, great efforts are still required. For example, because not all MoRFs adopt helical conformations [46,47], whether the non-helical MoRFs have preformed structures or anchor residues in these non-helical MoRFs also adopt bound-like conformations is unclear. It would also be valuable to experimentally test the side chain conformational preference observed in our simulations.

## 3. Method Section

### 3.1. Systems Setup

A series of simulations were performed based on the p53 *N*-terminal domain (Glu17–Asn29, p53N) (Table S1). Initial conformations for MD simulations were taken from the p53N-MDM2 complex (PDB ID 1YCR) [62] (trajectories MS1–MS10), the p53N-Taz2 complex (PDB ID 2K8F) [88] (trajectories TS1–TS5), or the disordered state of unbound p53N (trajectories DS1–DS5). The disordered states of unbound p53N were randomly selected snapshots ranging from 60 ns to 100 ns in the trajectory MS1. The duration for each trajectory varied between 20 and 100 ns (see Table S1). In the binding simulations, we randomly selected five conformations from the A1 group, B3 group (Figure 4), and the disordered state, respectively, as initial conformations of p53N; then we placed p53N close to the binding groove of MDM2 or Taz2 with the correct orientation (Figure 6). All binding simulations lasted for 10 ns.

### 3.2. Molecular Dynamics Simulations

The MD simulations were performed using the program GROMACS 4.07 [89,90] and the OPLS-AA/L force field [91]. The water molecules were modeled by the SPC/E representation [92]. Each of the starting conformations was placed in the center of a cubic water box with at least 10 Å from the box edge. Periodic boundary conditions were used. Counter ions (Na^+^ or Cl^−^) were added to neutralize the net charges. The long-range electrostatic interactions were treated with the particle mesh Ewald method [93]. The cutoff distances were set to 10 Å for short-range coulomb and van der Waals interactions. The bond lengths were fixed by the LINCS algorism [94], and a time step of 2 fs was used. Coordinates were saved every 5 ps.

Each system was first relaxed by 1000 steps of the steepest-descent energy minimization. After the minimization, the system was equilibrated at 300 K by 100 ps under an NVT ensemble and further equilibrated for 200 ps at constant pressure (1 bar). V-rescale [95] and Parrinello-Rahman [96] were used to couple the system to the simulation temperature and pressure with coupling constants of 0.1 ps and 2.0 ps, respectively. Production simulations were performed at constant temperature (300 K) and pressure (1 bar).

### 3.3. Analysis

Secondary structure analysis was assigned by the DSSP program [97]. NACCESS (http://wolf.bms.umist.ac.uk/naccess/) was used to calculate the SASA, using a default solvent probe radius of 1.4 Å. We monitored the side chain dihedral angles (χ_1_ and χ_2_) distribution of anchor residues in the helix state and disordered state. χ_1_ is defined by N-C_α_-C_β_-C_γ_ and χ_2_ is defined by C_α_-C_β_-C_γ_-C_δ__(1)_. Protein figures were produced by the VMD program [98].

## 4. Conclusions

In this study, we identified the anchor residues of the disordered p53N when binding to different targets, *i.e.*, Phe19, Trp23, and Leu26 for the p53N-MDM2 complex, and Leu22 for the p53N-Taz2 complex. From the all-atom simulations of the unbound p53N, we found that, as in ordered proteins, these anchor residues in p53N frequently sampled conformations similar to those in the complex states where anchor residues act as anchors, but seldom sampled those in the alternative complexes. We suggest that the bound-like conformations of anchor residues in the unbound state are an important factor in controlling the specific interaction between IDPs and their targets, in particular, stabilizing the transient encounter complexes. We also propose a new mechanism to account for the binding promiscuity of IDPs.

## Supplementary Materials

Figure S1. SASA analysis of the p53N in the disordered state (**a**,**c**) and the helix state (**b**,**d**) using the simulated trajectories. For comparison, the corresponding values of SASA in the complex states are presented.

Figure S2. Secondary structure analysis of p53N from p53N-MDM2 (**a**) and p53-Taz2 (**b**) during simulations. Simulations MS1–MS5 and TS1–TS5 are presented.

Figure S3. Schematic free energy landscape of p53N. The orange arrow indicates the relaxation of the initial complex structure to the helix state. The two green arrows indicate the transitions between the helix state and the disordered state.

**Table S1.** Details of the simulations.

Simulations	Initial Conformation ^*^	Simulation Duration (ns)	Unfolding Time (ns)
MS1	M	100	43
MS2	M	20	11
MS3	M	100	42
MS4	M	100	25
MS5	M	20	17
MS6	M	60	28
MS7	M	20	10
MS8	M	20	15
MS9	M	60	23
MS10	M	60	31
TS1	T	20	2
TS2	T	20	4
TS3	T	20	3
TS4	T	20	9
TS5	T	20	8
DS1	D	50	-
DS2	D	50	-
DS3	D	50	-
DS4	D	50	-
DS5	D	50	-

* M denotes the initial p53N structure from p53N-MDM2 complex; T for the initial p53N structure from p53N-Taz2 complex; D for the disordered state.

## Figures and Tables

**Table 1.** Population of bound-like conformations of anchor residues of the unbound p53N in different states.

**Complex compared**	**State of p53N**	Population of bound-like conformation *			
		Phe19	Leu22	Trp23	Leu26
p53N-MDM2	Helix	59.5%	–	62.4%	82.7%
	Disordered	19.2%	–	18.7%	76.2%
	Rotamer library	31.71%	3.65%	16.21%	62.52%
p53N-Taz2	Helix	12.2%	81.8%	–	–
	Disordered	6.0%	67.1%	–	–
	Rotamer library	47.08%	62.52%	5.32%	<1%

* “–” indicates the conformation in the bound state does not match a major group in the simulated χ_1_–χ_2_ distribution. Data for the rotamer library were adopted from Reference [65].
