# Supplementary figures and images for: Anchoring Intrinsically Disordered Proteins to Multiple Targets: Lessons from N-Terminus of the p53 Protein
Source: Int J Mol Sci. 2011 Feb 23;12(2):1410–30. doi: 10.3390/ijms12021410 (PMC3083713; doi:10.3390/ijms12021410)

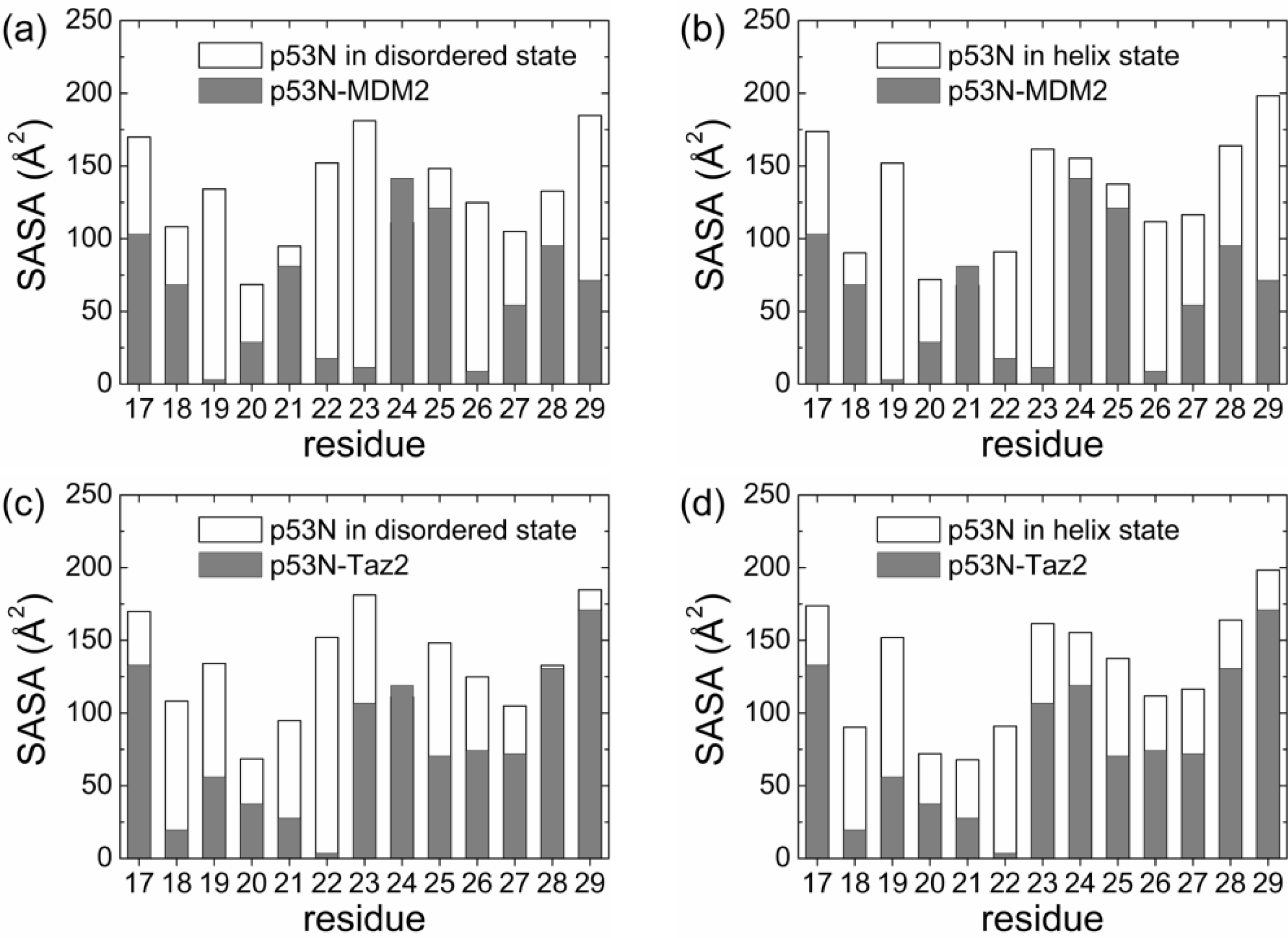

Supplement: Figure S1. [file ijms-12-01410-s001.tif]

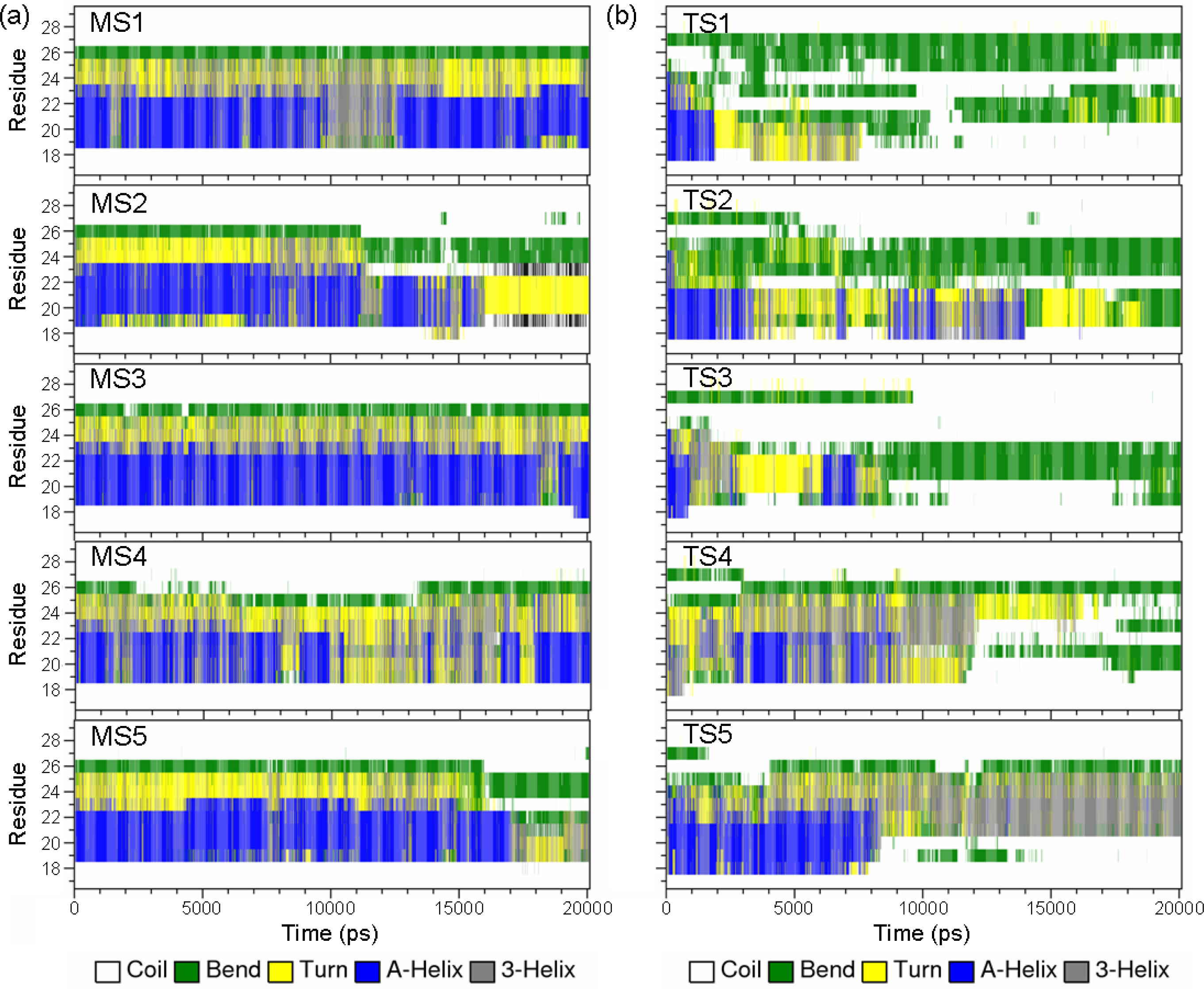

Supplement: Figure S2. [file ijms-12-01410-s002.tif]

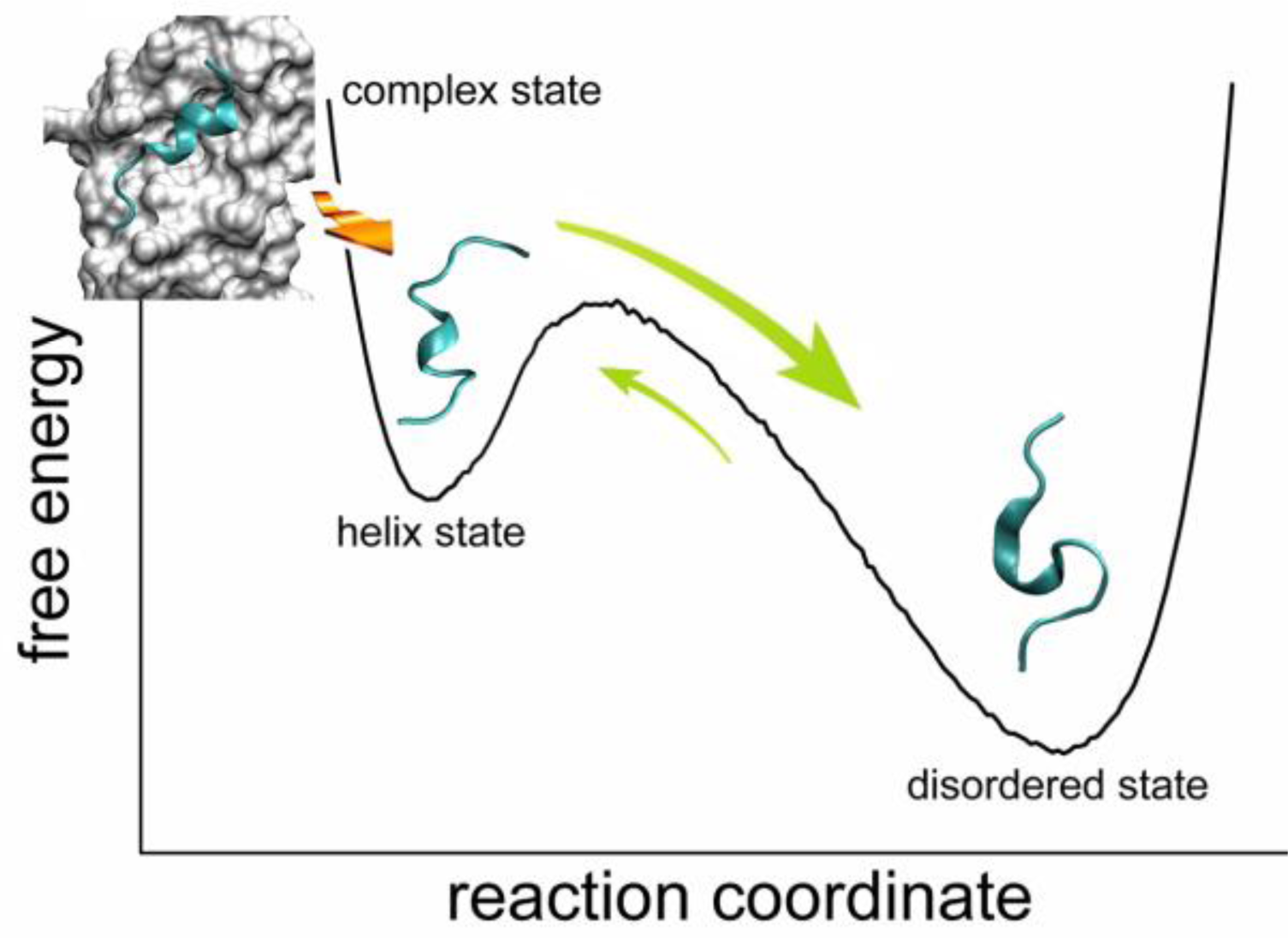

Supplement: Figure S3. [file ijms-12-01410-s003.tif]
